# Supplementary material for: Tead1a Initiates Transcriptional Priming Through the TEAD1a/YAP‐Notch1‐Spi1/Cebpα Axis to Promote Neutrophil Fate
Source: Adv Sci (Weinh). 2025 Aug 28;12(41):e05441. doi: 10.1002/advs.202505441 (PMC12591127; doi:10.1002/advs.202505441)
Supplement: Supplementary file 4 — Supplementary Dataset [file ADVS-12-e05441-s004.zip › 3-5.zip!/3-5/02-5.cluster/cluster12/Enrich/ego_MF.pdf]

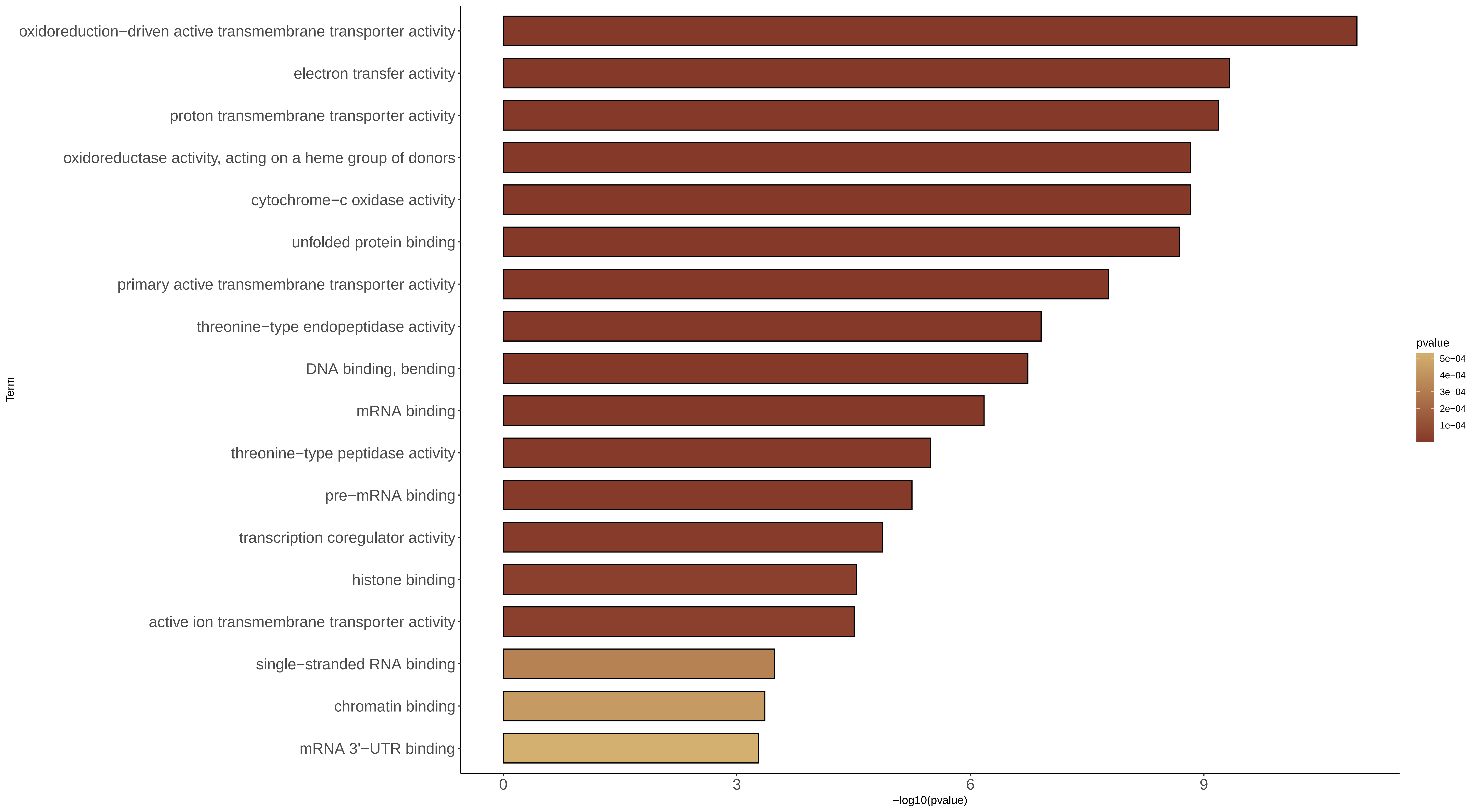
oxidoreduction−driven active transmembrane transporter activity
electron transfer activity
proton transmembrane transporter activity
oxidoreductase activity, acting on a heme group of donors
cytochrome−c oxidase activity
unfolded protein binding
primary active transmembrane transporter activity
threonine−type endopeptidase activity
DNA binding, bending
mRNA binding
threonine−type peptidase activity
pre−mRNA binding
transcription coregulator activity
histone binding
active ion transmembrane transporter activity
single−stranded RNA binding
chromatin binding
mRNA 3'−UTR binding
Term
0
3
6
9
−log10(pvalue)
pvalue
5e−04
4e−04
3e−04
2e−04
1e−04
